# Synergistic Neuroprotective Effects of Two Herbal Ingredients via CREB-Dependent Pathway

**DOI:** 10.3389/fphar.2016.00337

**Published:** 2016-09-27

**Authors:** Xu Liu, Dongxiao Wang, Runqing Zhao, Xianzhe Dong, Yuan Hu, Ping Liu

**Affiliations:** Department of Clinical Pharmacology and Pharmacy, Pharmacy Care Center, Chinese PLA General HospitalBeijing, China

**Keywords:** DISS, TFSA, CREB, synergistic effect, neuroprotective effect

## Abstract

As two natural oligosaccharide esters, 3,6’-Disinapoyl sucrose (DISS) and tenuifolisideA (TFSA) are originating from the root of *Polygala tenuifolia* Willd, a traditional Chinese medicine used in treatment of mental disorders. Previous reports have shown that both of them possess *in vitro* neuroprotective effects by stimulating different upstream pathways related with cyclic AMP-responsive element-binding protein (CREB). In the present study, we investigated the additive neuroprotective effects of DISS and TFSA on Glu-induced damage of SY5Y cells and purposed the possible underlying mechanism. The interaction between DISS and TFSA showed a clear-cut synergistic effect as evidenced by combination index (CI). Additional evidence from biochemical (NOS activity) assays confirmed their additive inhibition on the Glu-induced NOS hyperactivation. Moreover, we showed that co-treatment of DISS and TFSA resulted in an additively up-regulated phosphorylation of CREB as well as increased expressions of CRTC1 and BDNF. Neuroprotective effects of DISS and TFSA on Glu-induced decrease in cell viability were blocked by MAPK/ERK1/2 inhibitor (U0126) and PI3-K inhibitor (LY290042). Nevertheless, the CRTC1 or BDNF expression induced by these two compounds was significantly reduced in the presence of either ERK or PI3-K inhibitor, indicating that the two oligosaccharide esters shared some common pathways in the regulation of CREB-BDNF pathway. Taken together, we, for the first time, showed that DISS and TFSA exerted the additive neuroprotective effects on CREB-BDNF signaling pathway through complementary mechanisms.

## Introduction

As a common mental disorder, depression is estimated to affect 350 million people in the world by 2030 (Mathers et al., 2008). Conventional medicines for many depressed patients address only low efficacy but with untoward side effects (Blumenthal et al., 2012). Chinese herbs have a very long history for the use in the treatment of depression with few side effects reported. Extract of Radix Polygalae (the root of Polygala tenuifolia Willd) has been used as an antidepressant, a tranquilizer and an antipsychotic agent in the past several years (Chang and But, 1986; Hu et al., 2009; Liu et al., 2010). 3,6’-Disinapoyl sucrose (DISS) and tenuifoliside A (TFSA) are the principle active ingredients in the extract of Radix Polygalae. Our previous studies have indicated that both of DISS and TFSA have neuroprotective effects in different *in vitro* models (Hu et al., 2010, 2011, 2012). In addition, both can stimulate the expression and phosphorylation of key proteins in the cAMP response element-binding protein (CREB) signaling pathway (Liu et al., 2010; Dong et al., 2014).

cAMP response element-binding protein with its upstream and downstream effectors is thought to play a role in the pathophysiology of depression as well as in efficacy of antidepressant treatment (Majumder et al., 2004; Nair and Vaidya, 2006; Sakamoto et al., 2011). A serial of activity-inducible kinases (such as protein kinase C, Ca2+/CaM-dependent kinases II and IV, protein kinase A, ribosomal S6 kinase, AKT, mitogen/stress-activated kinase, and MAPK kinase 2) can increase the phosphorylation of CREB (Ghosh et al., 1994; Ginty et al., 1994; Tan et al., 1996; Hardingham et al., 1999; Li et al., 2011). Meanwhile, CREB-regulated transcription co-activators (CRTCs) can also dramatically increase CREB-mediated transcriptional activity independently of the phosphorylation status of CREB (Reichardt, 2006). Both of the above-mentioned mechanisms upregulate the downstream neurotrophic factor (BDNF) and affect neuronal proliferation, synaptic function, and synaptic plasticity.

Synergy may occur when two or more herbal ingredients are combined, which can mutually enhance each other’s effect when compared with administration alone (Ma et al., 2009). Therefore, we combined DISS and TFSA to determine if such a combination produced an additive or synergistic neuroprotective effect.

In the present study, we specifically aimed to assess the combination effect of two compounds, DISS and TSFA, in mediating neuroprotective effects *in vitro* and to further explore their additive mechanism via CREB pathway [CRTC1, pCREB (phosphor-CREB), BDNF]. Our goal was to investigate the potential usefulness of combining candidate active oligosaccharide esters for the treatment of mental disorders, by which we could reduce the risk of adverse effects by lowering their concentrations.

## Materials and Methods

### Materials

3,6’-Disinapoyl sucrose and TSFA were isolated from Radix Polygalae, identified by a combination of spectral methods (UV, IR, MS, and NMR) and purified by HPLC (purity >90%). U0126 and LY294002 were purchased from Selleck Chemicals LLC (Houston, TX, USA). Dulbecco’s modified Eagle’s medium (nutrient mixture F-12; DMEM/F-12) was obtained from Gibco (Carlsbad, CA, USA). Fetal bovine serum (FBS) was supplied by Hyclone (Logan, UT, USA). Polyclonal rabbit antibodies against BDNF, CREB, pCREB, CRTC1, and β-actin were all provided by Bioworld Technology (Atlanta, GA, USA) or Abcam (Burlingame, CA, USA).

### Cell Culture

SH-SY5Y human neuroblastoma cells were obtained from the American Type Culture Collection (Rockville, MD, USA) and cultured in DMEM/F-12 supplemented with 10% heat-inactivated FBS and 1% penicillin-steptomycin (50 U/mL). The cells were maintained in a humidified atmosphere with 5% CO_2_ at 37°C. The culture medium was refreshed every other day.

### Cell Viability Assay and Synergy Experiments

Cells were seeded into 96-well plates at a density of approximately 2 × 10^3^ cells/well and then grown for 24 h. Subsequently, cells were cultured with serum-free medium (0.1% BSA in growth medium without FBS) for another 24 h and then treated with DISS or TFSA at desired concentrations (15.625–2,000 μM). After 48-h treatment, cell viability was determined by Cell Counting Kit-8 (CCK-8; Keygen Biotech., Nanjing, China). Results were from six independent experiments, and each experiment was run in triplicate. The data were presented as percentage relative to untreated cells. The drug response curve was generated based on the determined relative cell viability, and 50% effective concentration (EC_50_) was measured using software Graphpad Prism5 (Roscilli et al., 2016). EC_50_ of combination was tested on TFSA at a fixed concentration (40 μM) plus DISS at various concentrations (600, 300, 150, 75, 37.5, 18.75 μmol/L). Combination effects of DISS and TFSA treatment were evaluated by calculating the combination index (CI) value as previously reported by Chou (Chou, 2006). CI = EC_50(DISSplusTFSA)_/EC_50 (DISSalone)_ + C_TFSA_/EC_50 (TFSAalone)_. C_TFSA_ was the TFSA concentration used in combination. In this analysis, synergism was defined as follows: CI > 1.1, antagonism; 1.1 < CI < 0.9, additive effect; 0.9 < CI < 0.3, synergic effect; and CI < 0.3, strong synergism.

### Inhibition Experiments

Briefly, cells were seeded into a 96-well plate or 6-well plate at a density of approximately 3 × 10^3^ cells/well or 1 × 10^4^ cells/well with full growth medium. After 24 h, medium was refreshed by serum-free medium and incubated 24 h. Cells were exposed to 8 mM glutamate (Glu) for 24 h, followed by treatment with DISS (75/150 μM), TFSA (25/50 μM) alone or combinations of DISS and TFSA at desired concentrations with or without antagonists (U0126/LY294002) for 24 or 48 h (assay BNDF mRNA, western blot), and each concentration of the inhibitor was established according to a previous study with minor modifications (Hu et al., 2014).

### Measurement of NOS and iNOS Activities

Cells were seeded into 6-well plates at a density of approximately 1 × 10^4^ cells/well cells and grown for 24 h. Subsequently, cells were exposed to 8 mM Glu for another 24 h. After different treatments for 48 h, cells were collected and re-suspended in PBS. Cell suspension was centrifuged, and the supernatant was collected into EP tubes and then stored at low temperature (0°C). NOS and iNOS activities as well as NO content were determined using a reagent kit (Nanjing Jiancheng Bioengineering Institute, Nanjing, China) using an ultraviolet spectrophotometer at a wavelength of 540 nm.

### BDNF Expression at the mRNA Level

Total RNA was extracted from cells using Trizol reagent. RNA concentration was determined using an Eppendorf BioPhotometer plus (Eppendorf, Germany). Subsequently, purified RNA was reversely transcribed into cDNA using iScript cDNA Synthesis Kit (Bio-Rad, Hercules, CA, USA) according to the manufacture’s instructions. BDNF expression at the mRNA level was assessed by qPCR using SsO Fast Eva Green Supermix (Bio-Rad, Hercules, CA, USA) according to the manufacturer’s instructions. GAPDH was selected as the housekeeping gene. The following sequences were used in the study:

BDNF up Primer: GACGGTCACAGTCCTAGAGAA,
BDNF low primer CCTTATGAATCGCCAGCCAAT;
GAPDH up primer ACTTCAACAGAGACACCCACTC,
GAPDH low primer TCTCTCTTCCTCTTGTGCTCTTGC.

Briefly, after an initial denaturation step at 95°C for 30 sec, amplifications were carried out with 40 cycle at a melting temperature of 95°C for 5 sec, an annealing temperature of 60°C for 5 sec, and an extension temperature of 65°C for 5 sec. Standard curves were generated for each gene, and the gene expression at the mRNA level was calculated relative to serial dilution of cDNA as described in Bio-Rad iQ5 System (California, USA). All experiments were conducted in triplicates. Relative expression was estimated using the 2^-ΔΔCt^ method.

### Western Blotting

After 48-h treatment, cells were collected and washed twice with ice-cold PBS. Subsequently, cell pellet was lyzed in lysis buffer (1% Triton, 20 mM Tris–HCl, 150 mM NaCl, 1 mM EDTA, pH 7.5) supplemented with one tablet of complete protease inhibitor cocktail (Roche, Laval, QC, Canada) and phosphatase inhibitor cocktail (Sigma, St. Louis, MO, USA), and the cell lysates were centrifuged at 1,000 *g* for 10 min at 4°C. Total amounts of proteins in each sample were determined by BCA kit, and the protein concentrations of all samples were adjusted to be the same. Supernatants containing 20 μg proteins were separated on 10% SDS-PAGE and electrotransferred onto polyvinylidenedifluoride (PVDF) membranes. Blots were blocked in Tris buffered saline containing 0.1% Tween-20 (TBST) and 5% non-fat-dried milk at room temperature for 1 h. Next, the membranes were incubated with the respective primary antibodies (anti-CREB 1:500, anti-phospho-CREB 1:500, anti-BDNF 1:2,000, and CRTC1 1:10,000) overnight at 4°C. Following three washes with TBST, the blots were incubated with the secondary horseradish peroxidase-conjugated antibody (1:6,000) at room temperature for 1 h, and optical density of protein bands was determined using an image analysis system (Bio-Imaging Analyzer, UVP). All the results of western blot consisted of three independent experiments, and each independent experiment was performed in triplicate.

### Statistical Analysis

Data were presented as mean ± SD. Comparisons were carried out using one way analysis of variance (ANOVA) followed by Tukey-Kramer’s test for *post hoc* analysis. All statistical analyses were performed using ANOVA by the PRISM software (GraphPad software, San Diego, CA, USA). *P* < 0.05 was considered as statistically significant.

## Results

### Combination of DISS and TFSA Inhibits Glu-Induced Decrease in Cell Viability

**FIGURE 1:**
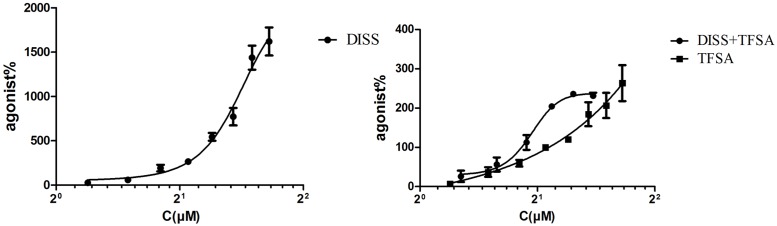
**EC_50_ of combination of DISS (3,6’-Disinapoyl sucrose) and TFSA (tenuifolisideA).** Cells were treated with DISS or TFSA at desired concentrations (15.625–2,000 μM) then cell viability were determined by cell counting kit-8. DISS or TFSA response curve was generated based on the determined relative cell viability, EC_50_ was measured. EC_50_ of combination was tested on TFSA at a fixed concentration (40 μM) plus DISS at various concentrations (600, 300, 150, 75, 37.5, 18.75 μmol/L). The EC_50_ of DISS, TFSA and the combination of DISS and TFSA was 606.4 ± 23.3 μM, 237.8 ± 13.3 μM, and 83.86 ± 1.06 μM, respectively. CI value of combination was 0.31 (CI < 1 means synergic effect).

**Figure 1** shows that the EC_50_ values of DISS and TFSA were 606.4 ± 23.3 μM and 237.8 ± 13.3 μM, respectively. The fixed concentration of 40 μM TFSA in combination was according to EC_20_ of TFSA (40.71 ± 2.47 μM). The EC_50_ of combination of DISS and TFSA was 83.86 ± 1.06 μM. CI value of co-treatment was 0.31, suggesting that combination of DISS and TFSA possessed a synergistic effect. Next, we investigated whether the combination of DISS and TFSA could have an additive or synergistic effect in preventing neuronal damage in SH-SY5Y cells. In order to lower the possible side effects of the compounds according to our previous experience, the concentrations of DISS and TFSA were set to 150/75 μM and 75/50 μM, respectively. The EC_20_ of DISS alone was 214.2 ± 1.23 μM. In combination, we chose 150 or 75 μM DISS that lower than EC_20_ of DISS and increased slightly TFSA concentrations based on EC_20_ of TFSA alone.

### The Effects of MAPK/ERK1/2 and PI3-K/Akt Inhibitors on the Protective Effects of DISS and TFSA against Glu-Induced Decrease in Cell Viability and Proliferation Rate

**FIGURE 2:**
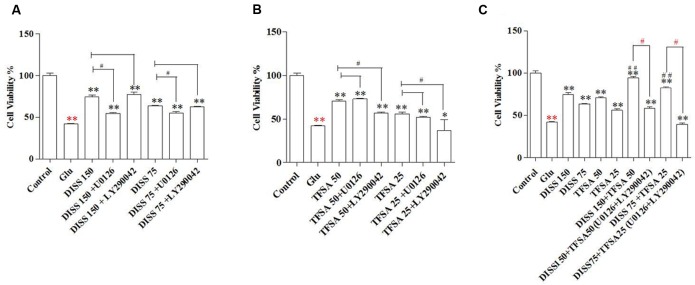
**Combination effect of DISS and TFSA on cell viability in SY5Y cells (*n* = 18).** SY5Y cells were exposed to 8 mM Glu for 24 h, followed by combination treatment of DISS and TFSA as well as inhibitors (U0126, LY294002). **(A)** Cells were treated by DISS and inhibitors. **(B)** Cells were treated by TFSA and inhibitors. **(C)** Cells were treated by combination of DISS, TFSA and inhibitors. ^∗^*p* < 0.05 and ^∗∗^*p* < 0.01 compared with the Glu group. Red ^∗∗^*p* < 0.01 compared with control group. ^#^*p* < 0.05, ^#^*p* < 0.001, and red ^#^*p* < 0.05 compared with its compound alone.

Based on this Glu-induced decrease in cell activity, the neuronal cytotoxicity of Glu could be partially inhibited by the pretreatment of DISS or TFSA in a dose-dependent manner. More importantly, the co-treatment of TFSA and DISS at two testing doses (75 μM + 25 μM and 150 μM + 25 μM) could produce a significant neuroprotective activity as compared with individual compounds alone, exhibiting increased cell viability from 48% to more than 85%. **Figures 2A,B** shows that such a protective effect of DISS at 150 and 75 μM could be blocked by the MAPK/ERK1/2 inhibitor, U0126 (10 μM), but not by the PI3-K inhibitor, LY290042 (20 μM). In contrast, the LY290042, but not U0126, inhibited the neuroprotective effect of TFSA. Moreover, the inhibitory effect of combination of U0126 and LY290042 became even more obvious, showing reduced cell viability from 85% to less than 60% (**Figure 2C**).

### Combination Effects of DISS and TFSA on the Expression of CREB Pathway Related Key Targets

**FIGURE 3:**
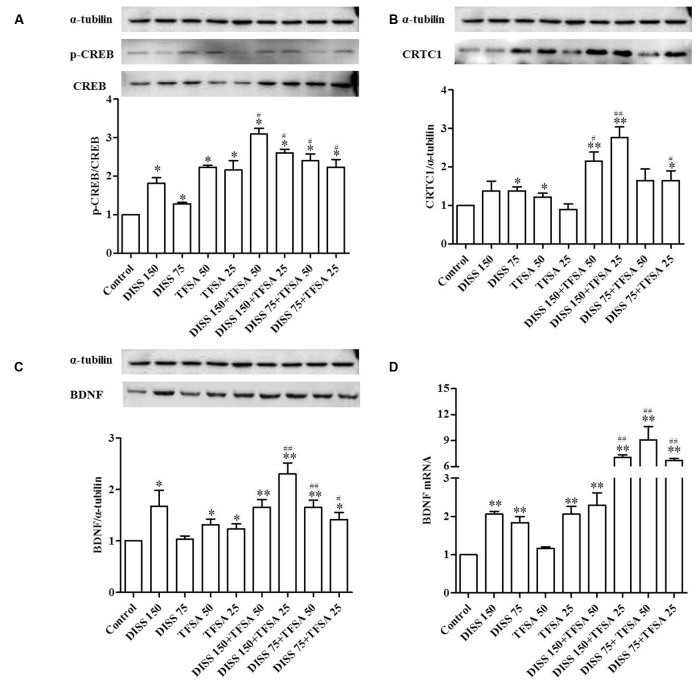
**Combination effect of DISS and TFSA on pCREB (phosphor-CREB)/CREB (cAMP response element-binding protein), BDNF, and CRTC1 expressions at the protein level as well as BDNF expression at the mRNA level in SY5Y cell (*n* = 18).** SY5Y cells were treated with 75 μM or 150 μM DISS, 25 μM or 75 μM TFSA, or combination of DISS and TFSA for 24 h. **(A,B,C)** Combination effect of DISS and TFSA on pCREB/CREB, CRTC1, and BDNF expressions at the protein level. **(D)** Combination effect of DISS and TFSA on BDNF expression at the mRNA level. ^∗^*p* < 0.05 and ^∗∗^*p* < 0.01 compared with the control group. ^#^*p* < 0.05 and ^##^*p* < 0.01 compared with its compound alone.

In order to further understand the mechanism of the combination effect of DISS and TFSA on the neuroprotection, we investigated the expressions of CREB, CRTC1, pCREB, and BDNF. DISS or TFSA alone could induce the protein expression of pCREB/CREB at both doses, and additive effect was observed for the combination of DISS and TFSA (**Figure 3A**). In addition, TFSA at 25 μM had no effect on the CRTC1 expression. However, combinations of 25 μM TFSA and 150 or 75 μM DISS both significantly increased the CRTC1 expression (**Figure 3B**). Meanwhile, the combinations of DISS and TFSA at different ratios all significantly enhanced the BDNF expression at the mRNA and protein levels (**Figures 3C,D**). Moreover, BDNF expression at the mRNA level was induced for more than threefold by combinations of 150 μM DISS and 25 μM TFSA; 75 μM DISS and 50 μM TFSA; or 75 μM DISS and 25 μM TFSA compared with compounds alone (**Figure 3D**).

### The Effects of ERK1/2 and PI-3K Inhibitors Are Involved in the Regulation of CRTC1 and BDNF Expressions by Induced by DISS and TFSA

**FIGURE 4:**
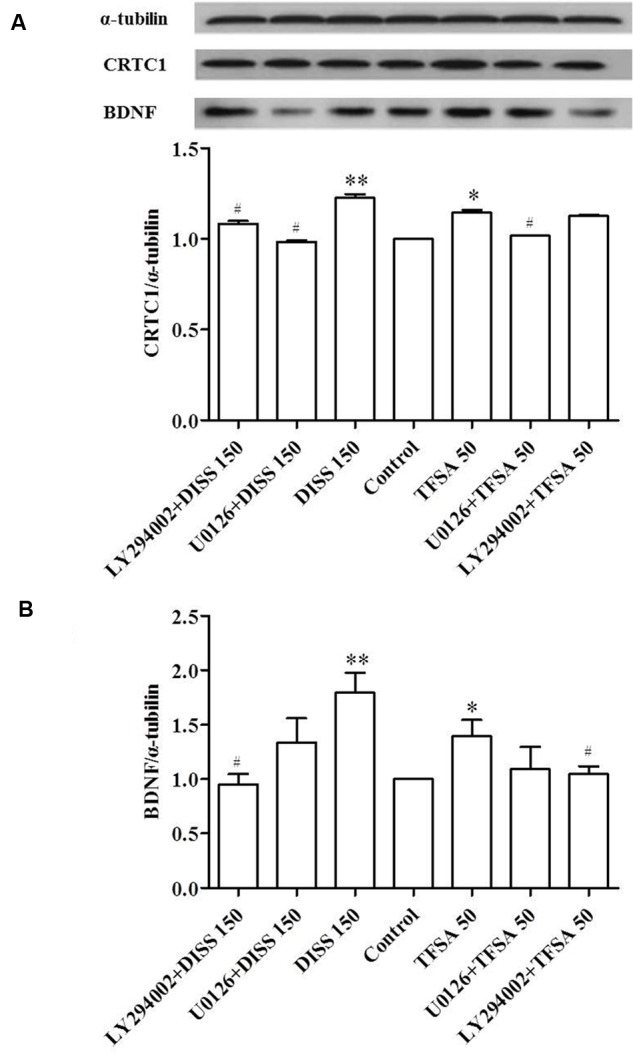
**Inhibitory effect of ERK1/2 and PI3K on DISS- or TFSA-induced BDNF and CRTC1 expressions in SY5Y cells (*n* = 9).** SY5Y cells were pretreated with 10 μM U0126 or 30 μM LY294002 for 30 min, followed by treatment with 150 μM DISS or 50 μM TFSA for 48 h. **(A)** Expressions of BDNF and CRTC1 were examined by western blotting. **(B)** The expression of BDNF and CRTC1. Data were expressed as mean ± SD. ^∗^*p* < 0.05 and ^∗∗^*p* < 0.01 compared with the untreated control group. ^#^*p* < 0.05 compared with its compound alone.

Considering the various CREB pathways activated by DISS and TFSA, we assessed the signaling mechanisms involved in CRTC1 and BDNF regulation using two inhibitors. **Figure 4** reveals that treatment of DISS or TFSA increased the expression of BDNF and CRTC1, but such an increase could be partially attenuated by either U0126 or LY294002. PI3-K inhibitor LY294003 showed a similar but weaker inhibitory effect on DISS-induced CRTC1 expression compared with U0126 (**Figure 4A**). The LY294002 decreased the DISS- or TFSA-induced BDNF expression by 47 and 25%, respectively. The ERK1/2 inhibitor U0126 decreased the DISS- or TFSA-induced BDNF expression by 25 and 21%, respectively. (**Figure 4B**). A bigger decrease in DISS- or TFSA-induced BDNF expression was caused by LY294003 compared with U0126 (**Figure 4B**).

### Combination Effects of DISS and TFSA on tNOS and iNOS Levels

**FIGURE 5:**
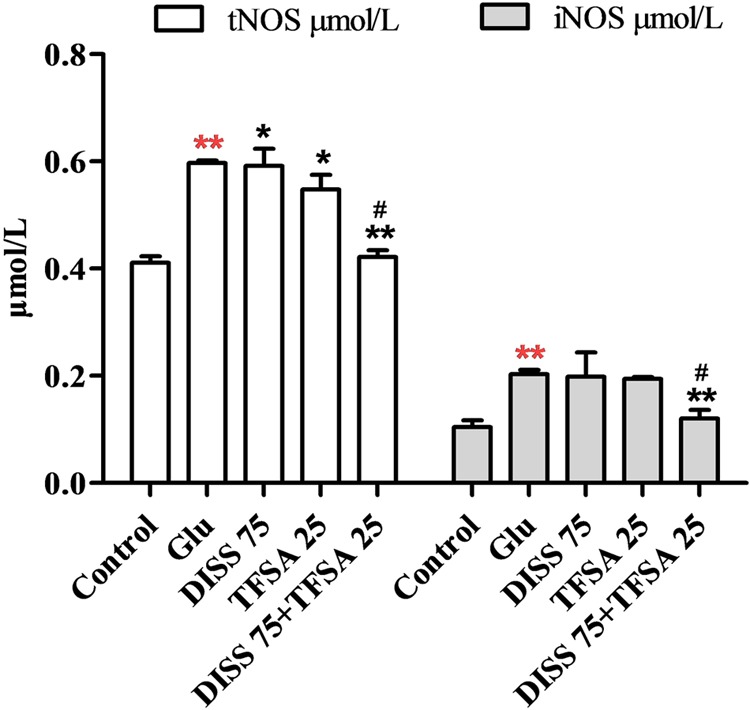
**Combination effect of DISS and TFSA on levels of tNOS and iNOS in SY5Y cells (*n* = 18).** SY5Y cells were exposed to Glu for 24 h, after which Glu was changed to DISS and TFSA combination medium for 48 h. Levels of tNOS and iNOS were determined by a reagent kit ^∗∗^*p* < 0.01 compared with Glu; ^∗∗^*p* < 0.01 compared with the control; ^∗^*p* < 0.05, ^#^*p* < 0.01 compared with its compound alone.

To further investigate the additive neuroprotection of DISS and TFSA against the Glu-induced cell injury, we examined the levels of tNOS and iNOS, which play an important role in cell apoptosis or neurodegenerative disorders (Chen et al., 2016; Zhu et al., 2016). **Figure 5** shows that Glu significantly increased the levels of tNOS and iNOS. DISS or TFSA treatment alone decreased the intracellular level of tNOS by 4–8%, but showed no effect on iNOS level. However, co-treatment of DISS and TFSA significantly decreased such an elevation of tNOS and iNOS levels by 29.3 and 40.5%, respectively. These results suggested that the combination of DISS and TFSA could more vigorously inhibit the Glu-induced NO overproduction.

## Discussion

The combination strategy offers a series of potential advantages, including less side effects and a more rapid or effective clinical response. In the current study, combination of the two active oligosaccharide esters demonstrated additive neuroprotective effects against Glu-induced damage. Compared with DISS or TFSA alone, their combination also significantly increased the expressions of pCREB, CRTC1, and BDNF.

Several studies have shown that DISS and TFSA play an antidepressant and neuroprotective role. DISS protects human neuroblastoma cells (SH-SY5Y) from Glu- or H_2_O_2_-induced damage, and DISS-mediated regulation of BDNF expression is associated with CREB-mediated transcription of BDNF and upstream activation of ERK1/2 and CaMKII (Hu et al., 2014). In our previous study, DISS possesses antidepressant-like properties, and its underlying mechanism might also be related to effects on hippocampal neuroplasticity gene BDNF (Hu et al., 2010). TFSA, with the similar chemical structure, can also promote the proliferation of C6 glioma cells, and its mechanism has been related to TrkB/BDNF/ERK and TrkB/BDNF/PI3-K signaling pathways (Dong et al., 2014).

Over the past decades, studies have suggested that neurotrophins and related signaling cascades are involved in the pathophysiology of depression. CREB upregulation may activate downstream targets, such as BDNF, after antidepressant treatment. In fact, a series of elegant studies have also shown that PI-3K/Akt and MAPK/Erk pathways, which can promote neuronal growth and survival, are two key signaling cascades regulating CREB/BDNF activation (Chen et al., 2007). Meanwhile, CRTC1, as another essential gene for long-term synaptic plasticity and synaptic development, also plays an important role in CREB-dependent gene transcription (Xue et al., 2015).

**FIGURE 6:**
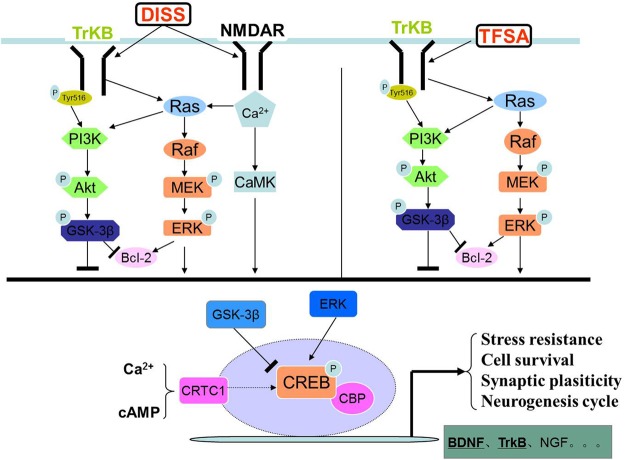
**The graph of DISS and TFSA synergistically activated the PI-3K/Akt and MAPK/ERK pathways**.

In the present study, we, for the first time, showed that DISS and TFSA possessed additive or synergistic protective effects on nerve regeneration *in vitro*. Additive effects were observed on cell viability, pCREB/CREB, BDNF, CRTC1, tNOS, and iNOS activities, synergistic effects were observed on BDNF mRNA. We adopted kinase inhibitors of U0126 and LY294002 to explore the mechanisms. It showed that combination of DISS and TFSA exerted an additive effect on CREB phosphorylation, and expressions of CRTC1 and BDNF at the protein level. **Figure 6** illustrates that DISS and TFSA activated the PI-3K/Akt and MAPK/Erk pathways, leading to stronger activation of the downstream CREB phosphorylation and simultaneously increased CRTC1 expression, and both of them were beneficial to the transcription of BDNF, which could eventually promote the neuronal survival.

In additional to the role in CREB/BDNF regulation, NO is an important messenger and effector molecule that possesses neurotransmitter and neuromodulator functions and participates in physiological and pathological activities. NO, together with iNOS and nNOS, is well known to be involved in the pathogenesis of neurodegenerative disorders (Chen et al., 2007; Di et al., 2010; Lin et al., 2014). Both N-methyl-D-aspartate receptor activation and Glu can induce the iNOS expression and NO production, subsequently leading to excessive damage caused by free radicals (Akama and Van Eldik, 2000; Di et al., 2010). Our results indicated that the levels of iNOS and tNOS were significantly increased in Glu-treated SY5Y cells, while combination of DISS and TFSA could ameliorate such Glu-induced NO production and iNOS overdosage, suggesting that the combination effect on neuroprotection was also involved in regulation of iNOS and tNOS activities.

## Conclusion

Combination of DISS and TFSA activated CREB-mediated MAPK and PI-3K cascades and induced the BDNF expression, revealing the underlying molecular mechanism of their additive neuroprotective effects. Our findings suggested the potential of a novel candidate oligosaccharide ester for the development of natural supplements against depression. Both DISS and TFSA are active oligosaccharide ester components found in the root of Polygala tenuifolia Willd. Our previous study showed that DISS has anti-depressive activity *in vivo* (Hu et al., 2010). The molecular weights of DISS and TFSA are 752 and 682 kDa, respectively. And both of them are fat-soluble. However, it remains unclear whether DISS, TFSA or its metabolites could pass the blood brain barrier. In our future study, we will further explore their additive or synergistic effects on mental disorder in animal models and investigate whether they can pass the blood brain barrier.

## Author Contributions

YH, XD and PL contributed in study design, YH, XL and RZ contributed in trial and writing. DW contributed in writing.

## Conflict of Interest Statement

The authors declare that the research was conducted in the absence of any commercial or financial relationships that could be construed as a potential conflict of interest.

## References

[B1] Akama K. T. Van Eldik L. J. (2000). Beta-amyloid stimulation of inducible nitric-oxide synthase in astrocytes is interleukin-1beta- and tumor necrosis factor-alpha (TNFalpha)-dependent, and involves a TNFalpha receptor-associated factor- and NFkappaB-inducing kinase-dependent signaling mechanism. *J. Biol. Chem.* 275 7918–7924. 10713108 10.1074/jbc.275.11.7918

[B2] Blumenthal J. A. Smith P. J. Hoffman B. M. (2012). Is Exercise a Viable Treatment for Depression? *ACSMs Health Fit. J.* 16 14–21. 23750100 10.1249/01.FIT.0000416000.09526.eb PMC3674785

[B3] Chang H. M. But P. P. H. (1986). *Pharmacology and Applications of Chinese Material Medica*. Toh Tuck Link: Word Scientific Publishing.

[B4] Chen L. N. Sun J. Yang X. D. Xiao K. Lv Y. Zhang B. Y. (2016). The brain NO levels and NOS activities ascended in the early and middle stages and descended in the terminal stage in scrapie-infected animal models. *Mol. Neurobiol.* 10.1007/s12035-016-9755-z [Epub ahead of print]. 26887380

[B5] Chen M. J. Nguyen T. V. Pike C. J. Russo-Neustadt A. A. (2007). Norepinephrine induces BDNF and activates the PI-3K and MAPK cascades in embryonic hippocampal neurons. *Cell. Signal.* 19 114–128. 10.1016/j.cellsig.2006.05.028 16876982

[B6] Chou T. C. (2006). Theoretical basis, experimental design, and computerized simulation of synergism and antagonism in drug combination studies. *Pharmacol. Rev.* 58 621–681. 10.1124/pr.58.3.10 16968952

[B7] Di X. Yan J. Zhao Y. Zhang J. Shi Z. Chang Y. (2010). L-theanine protects the APP (Swedish mutation) transgenic SH-SY5Y cell against glutamate-induced excitotoxicity via inhibition of the NMDA receptor pathway. *Neuroscience* 168 778–786. 10.1016/j.neuroscience.2010.04.019 20416364

[B8] Dong X. Z. Huang C. L. Yu B. Y. Hu Y. Mu L. H. Liu P. (2014). Effect of Tenuifoliside A isolated from *Polygala tenuifolia* on the ERK and PI3K pathways in C6 glioma cells. *Phytomedicine* 21 1178–1188. 10.1016/j.phymed.2014.04.022 24877714

[B9] Ghosh A. Ginty D. D. Bading H. Greenberg M. E. (1994). Calcium regulation of gene expression in neuronal cells. *J. Neurobiol.* 25 294–303. 10.1002/neu.480250309 7910846

[B10] Ginty D. D. Bonni A. Greenberg M. E. (1994). Nerve growth factor activates a Ras-dependent protein kinase that stimulates c-fos transcription via phosphorylation of CREB. *Cell* 77 713–725. 10.1016/0092-8674(94)90055-8 8205620

[B11] Hardingham G. E. Chawla S. Cruzalegui F. H. Bading H. (1999). Control of recruitment and transcription-activating function of CBP determines gene regulation by NMDA receptors and L-type calcium channels. *Neuron* 22 789–798. 10.1016/S0896-6273(00)80737-0 10230798

[B12] Hu Y. Li J. Liu P. Chen X. Guo D. H. Li Q. S. (2012). Protection of SH-SY5Y neuronal cells from glutamate-induced apoptosis by 3, 6’-disinapoyl sucrose, a bioactive compound isolated from Radix Polygala. *J. Biomed. Biotechnol.* 2012 1–5. 10.1155/2012/728342 21836813 PMC3151496

[B13] Hu Y. Liao H. B. Dai-Hong G. Liu P. Wang Y. Y. Rahman K. (2010). Antidepressant-like effects of 3, 6’-disinapoyl sucrose on hippocampal neuronal plasticity and neurotrophic signal pathway in chronically mild stressed rats. *Neurochem. Int.* 56 461–465. 10.1016/j.neuint.2009.12.004 20018220

[B14] Hu Y. Liao H. B. Liu P. Guo D. H. Rahman K. (2009). A bioactive compound from *Polygala tenuifolia* regulates efficiency of chronic stress on hypothalamic-pituitary-adrenal axis. *Pharmazie* 64 605–608. 19827305

[B15] Hu Y. Liu M. Liu P. Guo D. H. Wei R. B. Rahman K. (2011). Possible mechanism of the antidepressant effect of 3, 6’-disinapoyl sucrose from *Polygala tenuifolia* Willd. *J. Pharm. Pharmacol.* 63 869–874. 10.1111/j.2042-7158.2011.01281.x 21585386

[B16] Hu Y. Liu M. Y. Liu P. Dong X. Boran A. D. (2014). Neuroprotective effects of 3, 6’-disinapoyl sucrose through increased BDNF levels and CREB phosphorylation via the CaMKII and ERK1/2 pathway. *J. Mol. Neurosci.* 53 600–607. 10.1007/s12031-013-0226-y 24488601

[B17] Li X. Y. Zhan X. R. Liu X. M. Wang X. C. (2011). CREB is a regulatory target for the protein kinase Akt/PKB in the differentiation of pancreatic ductal cells into islet beta-cells mediated by hepatocyte growth factor. *Biochem. Biophys. Res. Commun.* 404 711–716. 10.1016/j.bbrc.2010.12.048 21156158

[B18] Lin S. E. Wu F. L. Wei M. F. Shen L. J. (2014). Depletion of arginine by recombinant arginine deiminase induces nNOS-activated neurotoxicity in neuroblastoma cells. *Biomed. Res. Int.* 2014:589424 10.1155/2014/589424 PMC4122191 25126568

[B19] Liu P. Hu Y. Guo D. H. Wang D. X. Tu H. H. Ma L. (2010). Potential antidepressant properties of Radix Polygalae (Yuan Zhi). *Phytomedicine* 17 794–799. 10.1016/j.phymed.2010.01.004 20541923

[B20] Ma X. H. Zheng C. J. Han L. Y. Xie B. Jia J. Cao Z. W. (2009). Synergistic therapeutic actions of herbal ingredients and their mechanisms from molecular interaction and network perspectives. *Drug Discov. Today* 14 579–588. 10.1016/j.drudis.2009.03.012 19508920

[B21] Majumder S. Varadharaj S. Ghoshal K. Monani U. Burghes A. H. Jacob S. T. (2004). Identification of a novel cyclic AMP-response element (CRE-II) and the role of CREB-1 in the cAMP-induced expression of the survival motor neuron (SMN) gene. *J. Biol. Chem.* 279 14803–14811. 10.1074/jbc.M308225200 14742439 PMC1761111

[B22] Mathers C. Fat D. Boerma J. (2008). *The Global Burden of Disease: 2004 Update*. Geneva: World Health Organization.

[B23] Nair A. Vaidya V. A. (2006). Cyclic AMP response element binding protein and brain-derived neurotrophic factor: molecules that modulate our mood? *J. Biosci.* 31 423–434. 10.1007/BF02704114 17006024 PMC4820646

[B24] Reichardt L. F. (2006). Neurotrophin-regulated signalling pathways. *Philos. Trans. R. Soc. Lond. B Biol. Sci.* 361 1545–1564. 10.1098/rstb.2006.1894 16939974 PMC1664664

[B25] Roscilli G. De Vitis C. Ferrara F. F. Noto A. Cherubini E. Ricci A. (2016). Human lung adenocarcinoma cell cultures derived from malignant pleural effusions as model system to predict patients chemosensitivity. *J. Transl. Med.* 14:61 10.1186/s12967-016-0816-x PMC4772534 26928703

[B26] Sakamoto K. Karelina K. Obrietan K. (2011). CREB: a multifaceted regulator of neuronal plasticity and protection. *J. Neurochem.* 116 1–9. 10.1111/j.1471-4159.2010.07080.x 21044077 PMC3575743

[B27] Tan Y. Rouse J. Zhang A. Cariati S. Cohen P. Comb M. J. (1996). FGF and stress regulate CREB and ATF-1 via a pathway involving p38 MAP kinase and MAPKAP kinase-2. *EMBO J.* 15 4629–4642. 8887554 PMC452194

[B28] Xue Z. C. Wang C. Wang Q. W. Zhang J. F. (2015). CREB-regulated transcription coactivator 1: important roles in neurodegenerative disorders. *Sheng Li Xue Bao* 67 155–162. 25896045

[B29] Zhu T. Yao Q. Wang W. Yao H. Chao J. (2016). iNOS induces vascular endothelial cell migration and apoptosis via autophagy in ischemia/reperfusion injury. *Cell Physiol. Biochem.* 38 1575–1588. 10.1159/000443098 27082814

